# Strength Performance Across the Oral Contraceptive Cycle of Team Sport Athletes: A Cross-Sectional Study

**DOI:** 10.3389/fphys.2021.658994

**Published:** 2021-07-01

**Authors:** Astrid Reif, Barbara Wessner, Patricia Haider, Harald Tschan, Christoph Triska

**Affiliations:** Subunit Sports Medicine, Exercise Physiology and Prevention, Centre for Sport Science and University Sports, Institute of Sport Science, University of Vienna, Vienna, Austria

**Keywords:** hormone pill, withdrawal bleeding, athletes, maximal voluntary force, hormonal contraceptive, female strength, exercise performance

## Abstract

Oral contraceptive pills (OCP) are very popular in female athletes not only for contraceptive effects but also due to the possibility of cycle manipulation. Moreover, it is debatable whether the manipulation of the menstrual cycle has a beneficial effect on exercise performance. Therefore, the aim of this study was to investigate potential differences in knee-extensor and flexor strength performance of first division team sport athletes between phases of the oral contraceptive cycle. Sixteen female handball players (age: 23.3 ± 3.1 years; body mass: 67.0 ± 8.52 kg; body stature: 1.68 ± 0.05 m) using a monophasic OCP participated in strength performance tests, once during OCP consumption (CONS) and once during withdrawal (WITH). Tests were performed on a dynamometer to measure knee-extensor and flexor maximal voluntary isokinetic and isometric torque. Prior to each test, body mass was assessed, and venous blood samples were collected. Wilcoxon signed-rank test and magnitude-based inferences have been conducted to analyze differences between WITH and CONS. Significance was accepted at *P* < 0.05. No significant differences between oral contraceptive cycle phases of knee-extensor and flexor strength parameters and body mass have been indicated (all at *P* > 0.05). Follicle-stimulating hormone (FSH) (*P* = 0.001) and luteinizing hormone (*P* = 0.013) were significantly higher in WITH, whereby estradiol and progesterone showed no significant difference between phases (both at *P* > 0.05). These results support the notion that knee-extensor and flexor isokinetic and isometric strength performance does not differ between phases of oral contraceptive cycle in well-trained team sport athletes. OCP intake is suggested to cause a stable but downregulated hormone cycle, which has no effect on knee-extensor and flexor strength when comparing oral contraceptive cycle phases. Therefore, manipulation of the female cycle using OCP in order to achieve a higher knee-extensor and flexor strength performance does not seem to be justified; however, it is currently unclear if cycle manipulation might affect other physiological systems.

## Introduction

Female athletes are often neglected in exercise physiological research; still, a growing body of literature recognizes the importance of research on the effects of the female cycle (Oleka, 2020). Approximately half of female elite athletes are using hormonal contraceptives, and the most commonly used type is the combined oral contraceptive pill (OCP) containing estrogen and progesterone (Prog) (Martin et al., 2018). OCP intake is popular, as it allows manipulation of the female cycle besides its contraceptive effect. The female cycle might be manipulated by extending the phase of pill intake and/or shortening the phase of withdrawal (Sulak et al., 2002). Cycle manipulation allows to control the timing of the withdrawal bleeding and potential painful side effects of menses.

The combination of estrogen and Prog in the combined OCP enables prevention of pregnancy and control of menstrual bleeding (Cooper and Mahdy, 2020). Its application is divided into two phases: 21 days of pill consumption (CONS) and 7 days without pill consumption, when withdrawal bleeding is induced (WITH) (Sims and Heather, 2018). The intake of exogenous estrogen and Prog through combined OCPs leads to changes in the cervix and a constantly low level of endogenous estrogen (E2) and Prog (Cooper and Mahdy, 2020). In detail, exogenous Prog leads to a lower level of gonadotropin-releasing hormone and consequently a minor secretion of follicle-stimulating hormone (FSH) and luteinizing hormone (LH). This effect inhibits the follicle development and suppresses the secretion of E2. When taking OCPs, exogenous estrogen supports this hormonal downregulation by slowing down FSH secretion (Cooper and Mahdy, 2020).

Interestingly, in physically active and competitive females, the manipulation of female cycle using OCP is widespread (Sulak et al., 2002). In elite athletes using OCPs, 72.6% reported to have their menstrual cycle manipulated at least once in the previous year. However, it is still questionable whether OCP-induced cycle manipulation is justified when it comes to maximizing athletic performance. Moreover, it is not completely clear whether consumption of exogenous hormones might affect strength performance when comparing different phases of the OCP cycle.

Previous studies assessing the effects of OCPs on exercise performance are controversial. On the one hand, it has been investigated that differences in strength performance through oral contraceptive cycle are unlikely (Elliott et al., 2005; Ekenros et al., 2013; Elliott-Sale et al., 2020). This is due to the low and stable concentrations of E2 and Prog. This might result in a constant strength performance through OCP cycles (McNulty et al., 2020). On the other hand, previous work suggests a potential benefit on sporadic cycle manipulation (Schaumberg et al., 2018). A previous study in OCP users found that drop jump performance was significantly lower in the late withdrawal phase. However, other jumping and sprinting tests of the same study did not support this notion (Rechichi and Dawson, 2009). Further, different types of exercises are suggested to bias the results and lead to controversial findings (Elliott-Sale et al., 2020). Therefore, an objective of this study was to investigate isolated thigh strength parameters using a dynamometer to reduce a potential bias of complex test performances and requirements.

Most studies investigated heterogeneously trained samples (Ekenros et al., 2013), untrained participants (Drake et al., 2003; Elliott et al., 2005; Mackay et al., 2019), or moderately trained participants (Lebrun et al., 2003; Bell et al., 2011). To provide a useful guidance, this study aims to provide new insights into a homogenously trained sample of first division team sport athletes using OCP during the general preparatory phase. Therefore, the purpose of this study was to narrow this research gap by assessing knee-extensor and flexor strength performance in team sport athletes by comparing strength performance across phases of OCP intake and withdrawal. We hypothesized non-significant differences in knee-extensor and flexor strength parameters between OCP cycles.

## Materials and Methods

### Participants

A sample of 16 female team sport athletes (age: 23.3 ± 3.1 years; body mass: 67.0 ± 8.5 kg in withdrawal phase; body stature: 1.68 ± 0.05 m) were recruited for this study. Participants were not conducting additional resistance training nor basic endurance sessions besides their habitual handball-specific training sessions. Inclusion criteria were (a) to play in the first Austrian handball league, (b) training at least three times a week, (c) a handball-specific training history of more than 3 years, and (d) using a commercially available low- to middle-dose monophasic OCP for at least half a year according to the instructions of the package insert. Monophasic OCPs contained 0.020–0.035 mg of ethinylestradiol (exogenous estrogen) and 0.10–2.00 mg of gestodene (exogenous Prog) (see Supplementary Material). The administration of the monophasic OCP consists of 7 days of no treatment (induction of withdrawal bleeding) (WITH) followed by 21 days of pill consumption (CONS). Timing of OCP consumption was not standardized to enable a common, regular daily routine of participants. Further, participants were free from any diseases and injuries and were included in the study only after medical approval.

This study has been approved by the host institutiońs Ethics Committee (#00435) and conformed to the principles of the World Medical Association’s Declaration of Helsinki (2013). After being fully informed about all procedures and risks of the study, participants provided written informed consent to participate.

### Experimental Approach to the Problem

The present study is designed as a cross-sectional study analyzing knee-extensor and flexor strength performance of female team sport athletes using OCPs. Oral contraceptive cycle phases are usually identified from the first day of withdrawal bleeding to the day preceding the next withdrawal bleeding. To investigate potential effects of oral contraceptive cycle phases (i.e., different concentrations in oral contraceptive cycle hormones) on knee-extensor and flexor strength, participants of this study reported twice to the laboratory to perform isokinetic and isometric maximal strength tests. Participants were tested a) at day 2 or 3 of the oral contraceptive cycle during withdrawal of OCP intake (WITH) and b) at day 16 or 17 of the oral contraceptive cycle during phase of OCP consumption (CONS), when OCP intake was reinitiated since at least 10 days. The tests were performed at the same time of the day, and the order of lab visits (i.e., WITH vs. CONS) was alternated to avoid potentially negative effects. Athletes were asked to avoid strenuous exercise 24 h prior to each testing session and to arrive at the laboratory in a fed and fully hydrated state. Moreover, they were required to refrain from alcohol the preceding 24 h and from caffeine or sports drink intake the preceding 3 h. Furthermore, subjects had to control their diet 48 h prior to testing and replicate the food intake for the second test. This was verbally verified prior to testing.

### Procedures

Body mass was measured during both visits to examine potential changes in body mass due to oral contraceptive cycle phases. Venous blood samples were collected on both test days in order to analyze for menstrual cycle hormones. The serum gel tube was centrifuged for 10 min at relative centrifugal force of 3,500 × g (Rotina 420R, Hettich, Vienna, Austria) after resting for 30 min. Samples of serum were frozen at –40°C until all samples were collected and were subsequently analyzed in a certified laboratory. Blood samples were collected to analyze for several female cycle hormones (i.e., FSH, LH, E2, and endogenous Prog) using Beckman Coulter Access Immunoassays (Beckman Coulter Inc., Brea, CA, United States). Prior to strength assessment, participants completed a standardized warm-up on a stationary ergometer (Racer 9, Kettler Freizeit GmbH, Ense-Parsit, Germany) for 10 min at a pre-defined workload of 0.75 W/kg body mass. To measure maximal voluntary knee-extensor and flexor strength performance, a dynamometer (ISOMED2000, Ferstl GmbH, Regensburg, Germany) with a sampling rate of 200 Hz was used. Participants were given a sufficient amount of time to familiarize with the device and the specific movements using submaximal contractions (Potzelsberger et al., 2015). Prior to formal data collection, maximal sessions for familiarization purposes were carried out. Unpublished data for maximal contractions from our laboratory for isokinetic and isometric strength on this dynamometer have demonstrated intraclass correlation coefficients of 0.88 < *r* < 0.99 and coefficients of variation (CV%) between 8.7 and 19.5%. However, if the difference between the first and second repetitions is greater than the CV%, an additional repetition would have been performed, but this was not required for any participant (*n* = 0). The device was combined with a unilateral knee attachment in order to measure knee-extensor and flexor strength. Maximal isokinetic and isometric torque were assessed in a seated position with the backrest at 75° for right and left legs separately. Participants were instructed to use the handles during contractions, and the straps were applied to minimize movement of the upper body during the test. All settings were recorded and replicated for the second visit.

Isokinetic strength was assessed by two or three identical concentric repetitions of knee joint flexion and extension for each leg separately. Angular velocity was set at 60°/s. The range of motion for the isokinetic tests was between 8° and 90°. A painless range of motion was ensured prior to each test. Passive rest for 3 min was provided between the repetitions. Analyzed parameters for isokinetic measures were (a) peak torque (PT) of extension, (b) PT of flexion, (c) H-Q ratio, and lateral deficit for (d) quadriceps muscles (LD_Q) and (e) hamstring muscles (LD_H). After another passive rest of 3 min, maximal isometric strength for extension was assessed. Therefore, two identical static repetitions of a 5-s maximal extension at 60° knee joint angle were performed, interspersed by another 3-min passive rest. Analyzed isometric parameters were (f) isometric PT, (g) peak rate of torque development (PRTD), and (h) time to PT (TTPT). The same procedures have been repeated on the other leg interspersed by 5-min passive rest. Temperature and humidity in the air condition-controlled laboratory were between 20 and 22°C, and between 45 and 55%, respectively.

### Statistical Analyses

Normality was assessed using the procedures of Shapiro–Wilk. Due to violation of normal distribution, a non-parametric Wilcoxon signed-rank test was applied to analyze differences in knee-extensor and flexor strength parameters and concentrations of cycle hormones between different phases of the oral contraceptive cycle. The effect sizes (*r*) were calculated by dividing the z-value by the square root of numbers of participants (trivial *r* < 0.01; small 0.01 ≤ *r* < 0.3; moderate 0.3 ≤ *r* < 0.5; large *r* ≥ 0.5). Magnitude-based inferences (MBIs) were conducted to determine the potential beneficial or harmful effects of OCP intake using a spreadsheet with compatibility limits set at 95%. Cohen effect size of 0.2 was used as the smallest worthwhile change (Hopkins, 2000). Quantitative changes of performance effects are presented through a qualitative descriptor, which has been assigned as follows: 0.5–5%, very unlikely; 5–25%, unlikely; 25–75%, possibly; 75–95%, likely; 95–99.5%, very likely; and > 99.5%, most likely (Batterham and Hopkins, 2006). Descriptive data are reported as median and interquartile range. Significance was accepted at an alpha level of *P* < 0.05. The analyses were conducted using SPSS statistical software package 26 (IBM SPSS Statistics, SPSS Inc., Chicago, IL, United States).

## Results

### Strength Parameters

**FIGURE 1:**
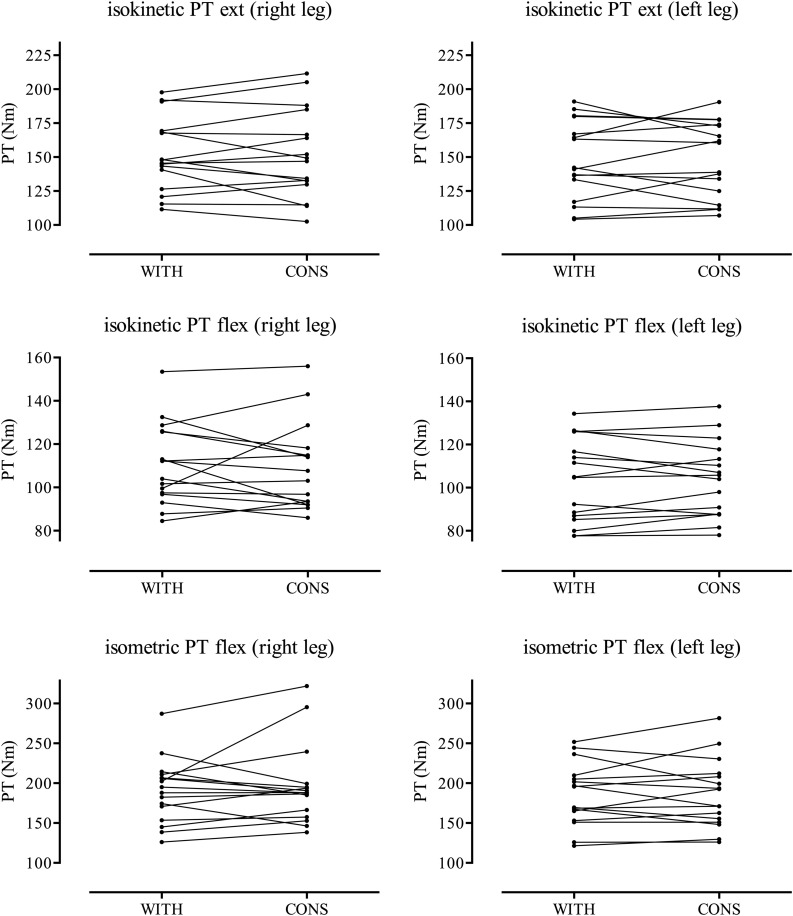
Individual changes of PT for extension and flexion for right and left legs during withdrawal phase (WITH) and consumption phase (CONS).

Descriptive data, results of the Wilcoxon rank test, and MBI calculations are presented in Tables 1, 2. The individual changes are displayed in Figure 1. Isokinetic parameters were unaffected by oral contraceptive cycle phases. PT extension, PT flexion, and H-Q ratio of right and left legs did not indicate significant differences with *trivial* effect sizes between cycle phases (Table 1). No significant differences were observed in LD_Q and in LD_H between oral contraceptive cycle phases demonstrating *trivial* to *small* effect sizes (Table 2). For isometric parameters, no significant differences between cycle phases have been detected. PT, PRTD, and TTPT right and left legs demonstrated no significant differences with *trivial* to *small* effect sizes (Table 1). MBI indicated no favor for any cycle phase, or effects were unclear, despite values for PT flexion right leg (uncertainly favors CONS) and PRTD right and left legs (uncertainly favors WITH).

**TABLE 1:** Descriptive data of isometric and isokinetic knee-extensor and flexor parameters comparing OCP withdrawal and consumption (*n* = 16).

		Isokinetic strength			Isometric strength		
		PT extension (Nm)	PT flexion (Nm)	H–Q ratio (%)	PT (Nm)	PRTD (Nm/s)	TTPT (ms)
Right leg	WITH	147 (130; 169)	108 (97; 126)	74 (69; 77)	192 (158; 210)	936 (767; 1,445)	410 (387; 465)
	CONS	148 (130; 180)	105 (92; 117)	72 (68; 79)	188 (160; 198)	1,218 (858; 1,481)	414 (351; 468)
	z	–0.08	–0.78	–0.39	–0.62	–1.45	–0.48
	*P*	0.938^T^	0.438^T^	0.697^T^	0.535^T^	0.148^S^	0.629^S^
	*% change WITH/trivial/CONS*	0.0/99.9/0.0	0.7/86.7/12.6	11.7/80.0/8.3	44.5/46.6/8.9	64.5/34.6/0.9	0.7/94.5/4.8
	*Qualitative inference*	No menstruation phase favored	Ambiguously favors CONS	Unclear	Unclear	Ambiguously favors WITH	No menstruation phase favored
Left leg	WITH	142 (121; 177)	105 (86; 124)	70 (63; 76)	183 (156; 209)	944 (675; 1,256)	449 (431; 468)
	CONS	150 (117;174)	105 (88; 117)	71 (65;75)	182 (152; 211)	1,314 (782; 1,384)	425 (388; 460)
	z	–0.05	–0.28	–0.10	–0.26	–1.65	–1.17
	*P*	0.959^T^	0.776^T^	0.917^T^	0.796^T^	0.100^S^	0.244^S^
	*% change WITH/trivial/CONS*	0.0/100.0/0.0	1.0/98.6/0.3	3.4/83.5/13.0	8.0/88.8/3.2	20.2/79.8/0.0	80.7/12.0/7.3
	*Qualitative inference*	No menstruation phase favored	No menstruation phase favored	Unclear	Unclear	Ambiguously favors WITH	Unclear

**Descriptive data are presented as med (IQR). WITH, withdrawal; CONS, consumption; PT, peak torque; PRTD, peak rate of torque development; TTPT, time to peak torque; H–Q ratio, hamstrings-quadriceps ratio; T, trivial effect size; S, small effect size; OCP, oral contraceptive pill; IQR, interquartile range.**

**TABLE 2:** Descriptive data of lateral deficit in isokinetic measurement comparing OCP withdrawal and consumption (*n* = 16).

	LD (%)	
	Hamstrings	Quadriceps
WITH	11.6 (5.5 14.1)	5.9 (2.3-10.9)
CONS	11.2 (3.5;14.4)	3.7 (8.1; 15.1)
Z	–0.72	–0.93
*P*	0.469^T^	0.352^S^
*% change WITH/trivial/CONS*	11.5/29.5/50.0	62.8/30.6/6.6
*Qualitative inference*	Unclear	Unclear

**Descriptive data are presented as med (IQR). WITH, withdrawal, CONS, consumption, LD, lateral deficit; T, trivial effect size; S, small effect; OCP, oral contraceptive pill.**

### Hormones and Body Mass

Descriptive data of blood parameters are depicted in Table 3. There were significant differences between oral contraceptive cycle phases in FSH and LH, both indicated by a *large* effect size. No significant differences between cycle phases were observed in Prog reflected by a *small* effect size. Concentrations for E2 were below the detection limit of 15 pg/ml in both phases. Furthermore, no significant differences in body mass have been found, and the effect size was *small*.

**TABLE 3:** Descriptive data of body mass and blood parameters comparing OCP withdrawal and consumption (*n* = 16).

	Body mass (kg)	FSH (IU/L)	LH (IU/L)	E2 (pg/ml)	Prog (ng/ml)
WITH	66.7 (59.7; 68.9)	5.65 (1.38; 9.73)	3.30 (0.40; 4.75)	<15	0.25 (0.10; 0.60)
CONS	66.6 (60.5; 69.7)	1.85 (0.25; 3.30)	1.05 (0.20; 2.68)	<15	0.25 (0.09; 0.60)
Z	0.31	–3.31	–2.48	n/a	–0.16
*P*	0.754^S^	0.001^L^	0.013^L^	n/a	0.875^S^

**Descriptive data are presented as med (IQR). WITH, withdrawal; CONS, consumption; FSH, follicle-stimulating hormone; LH, luteinizing hormone; E2, estradiol; Prog, progesterone; S, small effect size; L, large effect size; OCP, oral contraceptive pill.**

## Discussion

The aim of the present research was to examine potential differences in knee-extensor and flexor strength of female team sport athletes between oral contraceptive cycle phases. This study has not identified any physiologically meaningful differences in parameters reflecting knee-extensor and flexor strength performance between oral contraceptive phases. Another finding was that significantly higher concentrations of FSH and LH were found during OCP withdrawal, but no notable differences for E2 and Prog. The present results support our hypothesis that knee-extensor and flexor strength performance is not influenced by different oral contraceptive cycle phases in first division team sport athletes.

Maximum isokinetic and isometric strength performance in the present study did not significantly differ between oral contraceptive cycle phases with *trivial* to *small* effect sizes for all strength parameters analyzed. Further, these findings are supported by the MBI calculation, which indicated *unclear* effects or *no favor* for any oral contraceptive phase for H–Q ratio, TTPT, LD, and PT flexion left leg. In accordance with previous studies (Lebrun et al., 2003; Elliott et al., 2005; Rechichi and Dawson, 2009; Ekenros et al., 2013; Elliott-Sale et al., 2020), the present results indicate no physiologically meaningful differences between oral contraceptive cycle phases in knee-extensor and flexor strength performance of female athletes using OCPs. Interestingly, an *uncertain favor* was demonstrated for right leg PRTD and PT flexion for CONS and WITH, respectively. An explanation for this uncertain favor in PRTD might be caused by changes in muscle control patterns. Neuromuscular variation through contraceptive cycle has also been a suggested explanation for the differences in reactive strength of various drop jump heights in OCP users (Rechichi and Dawson, 2009). As the favor in the present results is uncertain and additionally no effects have been found in TTPT, we follow the suggestion that there is no physiologically meaningful effect on neuromuscular function by contraceptive cycle phases. However, it has to be taken into account that TTPT and PRTD are practical and functional parameters of neuromuscular function and of force production capacity, and no measures using the methods of electromyography or electrically stimulated contractions at different torque percentages of maximal voluntary contraction were carried out.

It is common knowledge that female cycle hormones have various physiological effects on athletes (e.g., Rechichi et al., 2009; Thompson et al., 2020). For example, E2 is suggested to cause an anabolic effect on skeletal muscle and lower blood lactate levels (Oosthuyse and Bosch, 2010). In contrast, Prog is suggested to have a synergetic catabolic effect (Oosthuyse and Bosch, 2010; McNulty et al., 2020) and may increase resting heart rate (Sedlak et al., 2012). Unsurprisingly, in the present work, concentrations of female cycle hormones have been low due to the downregulation of OCP. Concentrations of E2 and Prog were downregulated and were similar to OCP users in a previous study (Elliott et al., 2005). Unsurprisingly, E2 was even below the detection limit in some of the participants. Significantly lower concentrations of FSH and LH have been found in CONS compared with WITH, represented by a *large* effect size. It is, however, noteworthy that hormonal concentrations in both phases were apparently low enough to cause a concomitant suppressing effect on E2 and Prog secretions. In short, the analyzed endogenous hormones were merely low and stable; and in knee-extensor and flexor strength performance, no notable changes have been found through oral contraceptive cycle phases. Consequently, these findings confirm prior works that there are no influences of endogenous or exogenous hormones, which differently influence strength performance through an oral contraceptive cycle in elite athletes (Lebrun et al., 2003; Elliott et al., 2005; Rechichi and Dawson, 2009; Ekenros et al., 2013; Elliott-Sale et al., 2020).

For body mass, no significant differences between contraceptive cycle phases and merely a *small* effect size were found. Therefore, no changes in relative knee-extensor and flexor strength performance are evident. Participants of the present study were using OCPs for at least half a year. This finding goes along with several previous studies with participants consuming OCP regularly. Previous studies investigated weight gain and an increase in sum of skinfold only when females started a regular intake of OCPs (Lebrun et al., 2003).

Results of this study are limited to the fact that athletes were not using the same OCP product. This potential bias is alleviated since only athletes using a low- to middle-dose monophasic OCP with comparable concentration of ethinylestradiol and gestodene were recruited. Furthermore, monitoring more than one contraceptive cycle would have provided more detailed results. Due to the training routine and time issues of first division athletes, additional visits to the laboratory did not seem feasible for this study.

In conclusion, the current findings demonstrate no notable difference in knee-extensor and flexor strength performance through oral contraceptive phases. OCPs are a desirable option for many athletes due to the possibility to eliminate unpredictable menstruation and pain associated with eumenorrheic menstrual cycles (Elliott-Sale et al., 2020). Therefore, pain release and feeling more comfortable in competition cycle manipulation might have a beneficial influence on the individuals. From the perspective of isolated knee-extensor and flexor strength, there is no evidence of a benefit in female cycle manipulation in OCP users; however, our findings are limited to the effects of OCP on these muscle groups. Therefore, coaches and practitioners can take an informed decision that female cycle manipulation by using OCP does not seem to be justified when a higher knee-extensor and flexor strength performance is desired.

## Data Availability Statement

The original contributions presented in the study are included in the article/Supplementary Material, further inquiries can be directed to the corresponding author/s.

## Ethics Statement

The studies involving human participants were reviewed and approved by University of Vienna. The patients/participants provided their written informed consent to participate in this study.

## Author Contributions

AR conducted testing, analyzed the data, and wrote parts of manuscript. BW conducted statistical analysis and wrote parts of the manuscript. PH conducted testing and recruitment of participants. HT wrote parts of the manuscript and conceptualized the study. CT conceptualized the study and wrote parts of the manuscript. All authors contributed to the article and approved the submitted version.

## Conflict of Interest

The authors declare that the research was conducted in the absence of any commercial or financial relationships that could be construed as a potential conflict of interest.

## References

[B1] Batterham A. M. Hopkins W. G. (2006). Making meaningful inferences about magnitudes. *Int. J. Sports Physiol. Perform.* 1 50–57. 10.1123/ijspp.1.1.50 19114737

[B2] Bell D. R. Blackburn J. T. Ondrak K. S. Hackney A. C. Hudson J. D. Norcross M. F. (2011). The effects of oral contraceptive use on muscle stiffness across the menstrual cycle. *Clin. J. Sport Med.* 21 467–473. 10.1097/JSM.0b013e318230f50a 22008484

[B3] Cooper D. B. Mahdy H. (2020). *Oral Contraceptive Pills.* Treasure Island, FL: StatPearls.

[B4] Drake S. M. Evetovich T. Eschbach C. Webster M. (2003). A pilot study on the effect of oral contraceptives on electromyography and mechanomyography during isometric muscle actions. *J. Electromyogr. Kinesiol.* 13 297–301. 10.1016/s1050-6411(03)00024-5 12706609

[B5] Ekenros L. Hirschberg A. L. Heijne A. Friden C. (2013). Oral contraceptives do not affect muscle strength and hop performance in active women. *Clin. J. Sport Med.* 23 202–207. 10.1097/JSM.0b013e3182625a51 22948447

[B6] Elliott K. J. Cable N. T. Reilly T. (2005). Does oral contraceptive use affect maximum force production in women? *Br. J. Sports Med.* 39 15–19. 10.1136/bjsm.2003.009886 15618333 PMC1725011

[B7] Elliott-Sale K. J. McNulty K. L. Ansdell P. Goodall S. Hicks K. M. Thomas K. (2020). The effects of oral contraceptives on exercise performance in women: a systematic review and meta-analysis. *Sports Med.* 50 1785–1812. 10.1007/s40279-020-01317-5 32666247 PMC7497464

[B8] Hopkins W. G. (2000). *A New View on Statistics [Online]. Internet Society for Sport Science.* Available online at: http://www.sportsci.org/resource/stats/ (accessed January 17, 2021)

[B9] Lebrun C. M. Petit M. A. McKenzie D. C. Taunton J. E. Prior J. C. (2003). Decreased maximal aerobic capacity with use of a triphasic oral contraceptive in highly active women: a randomised controlled trial. *Br. J. Sports Med.* 37 315–320. 10.1136/bjsm.37.4.315 12893716 PMC1724664

[B10] Mackay K. González C. Zbinden-Foncea H. Peñailillo L. (2019). Effects of oral contraceptive use on female sexual salivary hormones and indirect markers of muscle damage following eccentric cycling in women. *Eur. J. Appl. Physiol.* 119 2733–2744. 10.1007/s00421-019-04254-y 31686212

[B11] Martin D. Sale C. Cooper S. B. Elliott-Sale K. J. (2018). Period prevalence and perceived side effects of hormonal contraceptive use and the menstrual cycle in elite athletes. *Int. J. Sports Physiol. Perform.* 13 926–932. 10.1123/ijspp.2017-0330 29283683

[B12] McNulty K. L. Elliott-Sale K. J. Dolan E. Swinton P. A. Ansdell P. Goodall S. (2020). The effects of menstrual cycle phase on exercise performance in eumenorrheic women: a systematic review and meta-analysis. *Sports Med.* 50 1813–1827. 10.1007/s40279-020-01319-3 32661839 PMC7497427

[B13] Oleka C. T. (2020). Use of the menstrual cycle to enhance female sports performance and decrease sports-related injury. *J. Pediatr. Adolesc. Gynecol.* 33 110–111. 10.1016/j.jpag.2019.10.002 31678355

[B14] Oosthuyse T. Bosch A. N. (2010). The effect of the menstrual cycle on exercise metabolism: implications for exercise performance in eumenorrhoeic women. *Sports Med.* 40 207–227. 10.2165/11317090-000000000-00000 20199120

[B15] Potzelsberger B. Stoggl T. Lindinger S. J. Dirnberger J. Stadlmann M. Buchecker M. (2015). Alpine Skiing With total knee ArthroPlasty (ASWAP): effects on strength and cardiorespiratory fitness. *Scand. J. Med. Sci. Sports* 25 Suppl 2 16–25. 10.1111/sms.12475 26083698

[B16] Rechichi C. Dawson B. (2009). Effect of oral contraceptive cycle phase on performance in team sport players. *J. Sci. Med. Sport* 12 190–195. 10.1016/j.jsams.2007.10.005 18054842

[B17] Rechichi C. Dawson B. Goodman C. (2009). Athletic performance and the oral contraceptive. *Int. J. Sports Physiol. Perform.* 4 151–162. 10.1123/ijspp.4.2.151 19567919

[B18] Schaumberg M. A. Emmerton L. M. Jenkins D. G. Burton N. W. Janse de Jonge X. A. K. Skinner T. L. (2018). Use of oral contraceptives to manipulate menstruation in young, physically active women. *Int. J. Sports Physiol. Perform.* 13 82–87. 10.1123/ijspp.2016-0689 28459358

[B19] Sedlak T. Shufelt C. Iribarren C. Merz C. N. (2012). Sex hormones and the QT interval: a review. *J. Womens Health (Larchmt)* 21 933–941. 10.1089/jwh.2011.3444 22663191 PMC3430484

[B20] Sims S. T. Heather A. K. (2018). Myths and methodologies: reducing scientific design ambiguity in studies comparing sexes and/or menstrual cycle phases. *Exp. Physiol.* 103 1309–1317. 10.1113/EP086797 30051938

[B21] Sulak P. J. Kuehl T. J. Ortiz M. Shull B. L. (2002). Acceptance of altering the standard 21-day/7-day oral contraceptive regimen to delay menses and reduce hormone withdrawal symptoms. *Am. J. Obstet. Gynecol.* 186 1142–1149. 10.1067/mob.2002.122988 12066088

[B22] Thompson B. Almarjawi A. Sculley D. Janse de Jonge X. (2020). The effect of the menstrual cycle and oral contraceptives on acute responses and chronic adaptations to resistance training: a systematic review of the literature. *Sports Med.* 50 171–185. 10.1007/s40279-019-01219-1 31677121

